# Data-Driven Real-Time Rice Milling Optimisation via YOLO26 Machine Vision and Adaptive Closed-Loop Motor Control

**DOI:** 10.3390/s26144557

**Published:** 2026-07-18

**Authors:** Benjamin Ilo, Yogang Singh, Hongwei Zhang

**Affiliations:** 1 Advanced Food Innovation Centre (AFIC), Sheffield Hallam University, Sheffield S9 2AA, UK; b.ilo@shu.ac.uk; 2 School of Engineering and Built Environment, Sheffield Hallam University, Sheffield S1 1WB, UK; y.singh@shu.ac.uk

**Keywords:** rice milling, YOLO26, closed-loop control, machine vision, PWM actuation, Industry 4.0, food quality inspection

## Abstract

Rice-milling quality is conventionally inspected post-process, leaving operators unable to correct breakage as it occurs. We present and quantitatively validate a cloud-mediated closed-loop architecture that couples a YOLO26 machine-vision pipeline to Arduino-based actuator control on a laboratory rice mill. The image-acquisition node uploads frames to a cloud repository; an inference and analysis node retrieves them, runs YOLO26 detection with a hybrid post-process classifier to estimate the broken-rice fraction, and issues a command to an Arduino microcontroller that drives PWM-modulated motor and vibrator actuators. The detector achieved a mean Average Precision of 0.951 (peak precision 0.99, peak recall 0.98) on a held-out test set of 100 images. In a matched comparison against an open-loop baseline (n=196,000 kernels, broken fraction 21.04%), closed-loop operation (n=114,000 kernels) reduced the broken fraction to 6.19%, an absolute improvement of 14.85 percentage points (two-proportion *z*-test: z=112.8, p<0.001, 95% CI for the absolute reduction: 14.62–15.08 pp). Dynamic analysis identified a near-linear plant gain of 1.0–1.5% breakage per 1% PWM, providing the empirical basis for future formal PID and Model Predictive Control synthesis. The principal empirical contribution is a quantitative characterisation of the PWM-to-breakage transfer relationship of a rice-milling actuator under deep-learning-derived quality feedback, together with a matched open-loop/closed-loop demonstration that this feedback loop moves the laboratory prototype from non-compliant to Grade A-equivalent quality at constant throughput. The lab-scale prototype is not yet industrial; a roadmap to pilot-scale deployment is outlined.

## 1. Introduction

### 1.1. Background and Motivation

Rice is the staple food of more than half of the world’s population and supplies over 21% of global caloric intake [1,2]. Milling quality, quantified by the proportion of whole kernels to broken fragments, is the principal determinant of commercial grade and consumer acceptance [3,4]. In commercial practice, the broken-rice fraction is determined by sieve analysis or operator inspection after milling, leaving no opportunity to correct the process while a batch is being milled. The economic consequence is substantial. A one-percentage-point increase in broken fraction can shift a batch from Grade A premium to a lower commercial grade with proportionally reduced market value [5,6].

#### Scope of This Study

The results reported below are obtained on a single rice cultivar under standardised laboratory illumination on a Rice Inspection Bed (R.I.B.). Extrapolation to industrial throughput, multi-varietal operation, and unstructured environments requires further validation, which is discussed as priority future work in Section 5.2.

### 1.2. Related Work

Existing efforts to address this problem fall into three broad categories.

Vision-only inspection systems.

The majority of published work applies machine-vision and deep-learning models to *post-process* grading of rice kernels [4,7,8]. Convolutional architectures including YOLO variants have been shown to classify whole and broken grains with accuracies above 95% [9,10,11]. While these systems are valuable for downstream sorting and grading, they do not influence the milling process itself, while quality degradation is detected only after it has occurred.

Control-only systems.

A second body of work has applied conventional process-control techniques, including PID and rule-based control, to milling parameters such as roller speed and feed rate [2,12]. Such systems regulate the actuators reliably, but the controlled variable is typically a proxy (motor current, throughput) rather than the quality of the milled product itself. The control loop is therefore blind to the very metric the operator cares about most.

Integrated vision-control systems.

A small number of recent studies have proposed coupling machine vision with actuator control for food processing [13]. However, reported integrations have generally been demonstrated for sorting or rejection mechanisms (binary actuation of an air jet or diverter) rather than for continuous modulation of the milling process. To the authors’ knowledge, no published study has quantitatively validated a closed-loop architecture in which a real-time deep-learning quality estimate continuously modulates the milling parameters.

### 1.3. Research Gap and Contributions

The literature reviewed in Section 1.2 reveals three specific gaps:

1. Existing rice-quality vision systems are open-loop with respect to the milling process. Quality is measured but not acted upon in real time.
2. Existing control systems for rice milling regulate process variables that are only indirectly related to product quality.
3. The newest end-to-end detection architectures, including YOLO26 [14], have not yet been evaluated in the context of real-time and closed-loop food-processing control.

The contributions of this paper are

1. A cloud-mediated closed-loop architecture for rice milling that decouples image acquisition, deep-learning inference, and actuator control across separate computational nodes (Section 2.2).
2. A quantitative comparison against a matched open-loop baseline on a combined sample of more than 310,000 classified kernels, demonstrating a 14.85 pp absolute reduction in broken-rice fraction (Section 3.4).
3. A quantitative characterisation of the PWM-to-breakage transfer relationship of the milling actuator, yielding an empirical plant gain $K^{*}\approx$ 1.0–1.5% breakage per 1% PWM (Section 3.5), which provides the design basis for future formal PI/PID/MPC controller synthesis. To our knowledge, this is the first published quantitative characterisation of this relationship for a rice-milling actuator under deep-learning-derived quality feedback.

## 2. Materials and Methods

Sample.

*Oryza sativa* grains were collected from an industrial milling facility and sieved to remove dust and fines, ensuring consistent morphological quality. Two classes were defined: Good (whole kernels, length ≥5.5 mm, no visible cracks) and Broken (fragments, length < 5.5 mm).

Image acquisition for the dataset.

**Figure 1:**
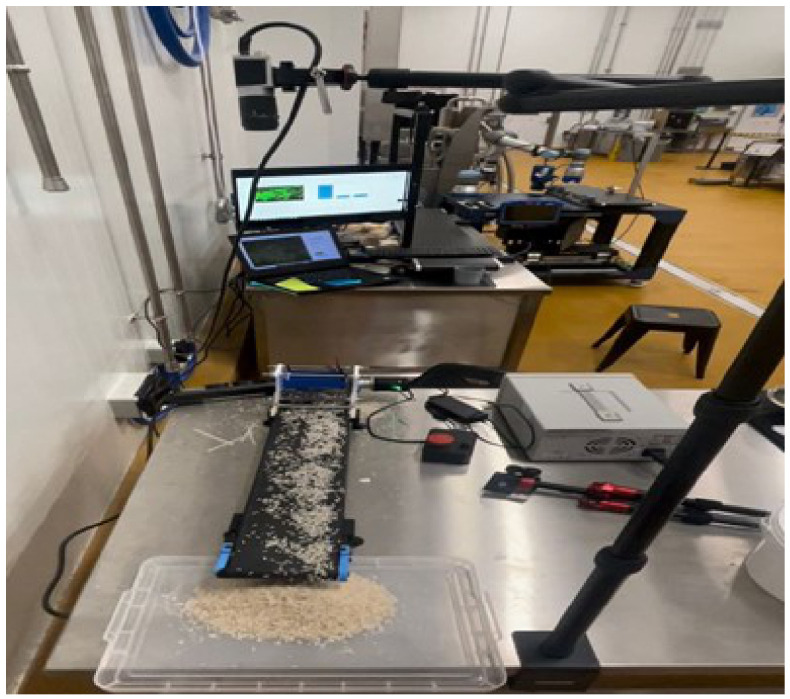
Dataset-processing pipeline.

A total of 1000 high-resolution images (4032×3024 px) were captured under standardised laboratory illumination as shown in Figure 1, with an average of approximately 70 kernels per image and grains arranged in random orientations on the Rice Inspection Bed (R.I.B., Section 2.1).

Annotation.

All kernels were annotated using Roboflow, with each grain enclosed in a tight axis-aligned bounding box. To ensure consistency, every image was re-inspected, and any boxes whose Intersection-over-Union (IoU) disagreement exceeded 0.10 were re-annotated. The single-object class *Rice_kernel* was used at the detection stage; the Good/Broken assignment is made downstream by the post-process classifier (Section 2.3), preserving sample efficiency by allowing the detector to focus on the easier kernel-localisation task.

Class balance.

The annotated dataset contained approximately 70,000 kernel-level instances, with a Good:Broken ratio of approximately 4:1 reflecting realistic milled-output composition. To prevent the post-process classifier from being biased toward the majority class, threshold parameters were calibrated on a separate validation subset rather than on the training data.

Data split.

The 1000 images were partitioned into **70% training (700 images), 20% validation (200 images) and 10% test (100 images)** using stratified sampling on the per-image broken-kernel proportion to ensure consistent class distributions across splits. All performance metrics reported in Section 3.1 are computed on the held-out test set.

### 2.1. Rice Inspection Bed and Experimental Setup

**Figure 2:**
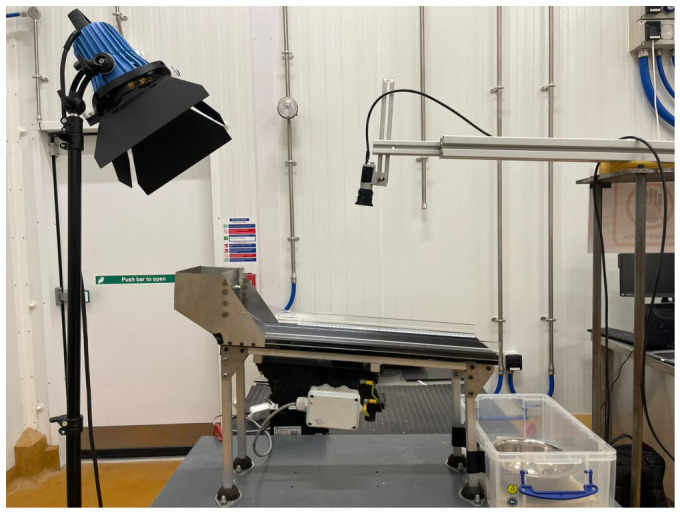
Rice Inspection Bed (R.I.B.) experimental setup showing the inclined Delrin platform, stainless-steel hopper, vibrator unit, GigE camera, and LED illumination.

Figure 2, a Rice Inspection Bed (R.I.B.) that was constructed from a 600×200×18 mm Delrin sheet with an anti-reflective matte black surface to suppress optical noise. A stainless-steel hopper was mounted at the rear, and the assembly was supported on four aluminium legs with a fixed forward incline (15–30°, adjustable). A variable-frequency vibrator (10–50 Hz) on the R.I.B. controls grain dispersion across the inspection surface; this vibrator is independent of the closed-loop actuators on the rice mill and was held at a fixed setting throughout. Images were captured by a GigE Vision CMOS camera (24 MP, global shutter, 25 mm macro lens) at 2 fps, with synchronised pulsed LED illumination through diffusers to ensure uniform lighting.

The 1000-image dataset was partitioned into training, validation, and test subsets using a stratified 70/20/10 image-level split so that no frame appears in more than one subset. Images span a range of broken-fraction densities representative of the milling variation observed under the tested operating regime, providing distributional coverage of the classifier’s decision boundary without relying on frame-level replication. All images were captured on the same single-cultivar dataset under the standardised laboratory illumination described above; multi-varietal and multi-illumination extension is identified as a priority direction in Section 5.2.

### 2.2. Closed-Loop System Architecture

**Figure 3:**
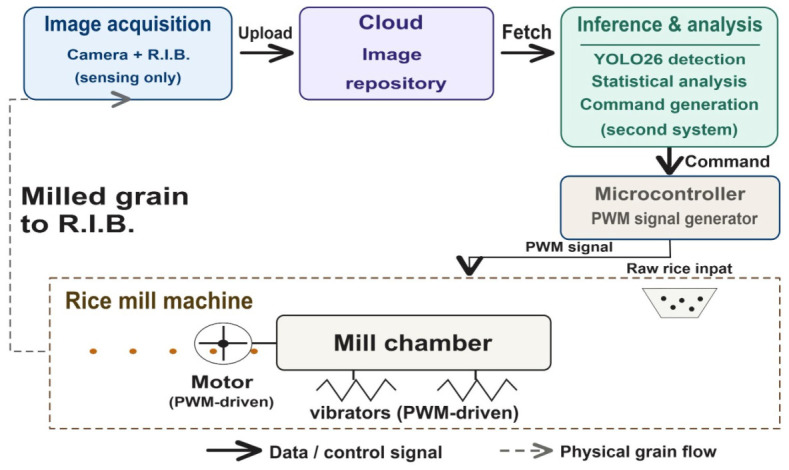
Representation of the closed-loop system architecture showing sequence of operation and direction of flow.

The integrated platform implements an explicit four-stage data flow (Figure 3):

1. **Capture:** The camera mounted above the R.I.B. captures successive frames of the milled grain stream and uploads each frame to a cloud repository over ethernet.
2. **Transfer:** The cloud repository serves as a shared data plane, decoupling the acquisition node from the compute node so that inference can scale independently of the sensing hardware.
3. **Inference:** A separate inference and analysis node retrieves the latest frame, runs YOLO26 detection followed by the post-process classifier (Section 2.3), computes the frame-level broken-rice fraction B(k), and issues a high-level command to the microcontroller.
4. **Control actuation:** An Arduino microcontroller converts the high-level command into a PWM signal at the actuation cadence, driving the motor and vibrators of the rice mill (Section 2.5).

### 2.3. YOLO26 Detection Pipeline

The detection pipeline is intentionally hybrid: a deep network performs kernel localisation, and a lightweight post-process classifier performs the Good/Broken assignment. The detection stage uses YOLO26, which was released by Ultralytics in September 2025 and introduces three architectural changes relevant to industrial deployment [14,15]: (i) an end-to-end prediction head that produces non-redundant bounding boxes without Non-Maximum Suppression (NMS) post-processing, eliminating dependence on hand-tuned NMS thresholds; (ii) removal of Distribution Focal Loss to simplify model export and edge deployment; and (iii) Small-Target-Aware Label Assignment (STAL) to improve detection of small or partially occluded objects. Reported benchmarks on Jetson Orin hardware indicate up to 43% reduction in CPU inference latency for the nano variant relative to YOLOv11 [15,16].

The post-process classifier assigns each detected bounding box to either Good or Broken on the basis of the kernel’s aspect ratio and contour area within the bounding box. This system design isolates the deep-learning component (localisation, which is hard) from the classification step (a simple post-process decision based on morphological features, easily auditable and adjustable per varietal). For each frame shown in Equation (1), *k* frame-level metrics are then computed: Good count $n_{g}\left(k\right)$, Broken count $n_{b}\left(k\right)$,

(1) $$B\left(k\right)=\frac{n_{b}\left(k\right)}{n_{g}\left(k\right)+n_{b}\left(k\right)}\times 100\%$$

Why YOLO26 specifically.

YOLO26 was selected over earlier YOLO variants (YOLOv8, YOLOv11, YOLOv12, YOLOv13) for three reasons grounded in the closed-loop requirement rather than peak benchmark scores. First, the NMS-free prediction head removes a hand-tuned threshold from the deployment loop, simplifying long-term operation by non-specialist plant staff. Second, the reduced CPU latency makes embedded edge inference feasible without dedicated GPU hardware, which is a non-trivial cost consideration for industrial rollout (Section 4.4). Third, the small-object-aware label assignment (STAL) directly addresses the most operationally difficult cases in our application, namely partially occluded grains in dense feed conditions.

### 2.4. YOLO26 Training and Detection Performance

The model was fine-tuned from the COCO-pretrained YOLO26 backbone using the standard Ultralytics training pipeline with mosaic augmentation, random flip, and colour jitter. Training proceeded for 100 epochs on a single workstation. Held-out test-set performance is reported in Section 3.1.

Operating confidence threshold.

A confidence threshold of τ=0.55 was selected for live operation. This value was chosen to balance two competing requirements specific to the closed-loop application: (i) sufficient recall to ensure that the broken-rice fraction estimate is not systematically biased by missed detections and (ii) sufficient precision to ensure that the control signal B(k) is not corrupted by spurious false positives. Inspection of the test-set Precision–Confidence and Recall–Confidence curves (Section 3.1) shows that this threshold sits at the plateau of precision while retaining ∼0.81 recall, providing stable control behaviour without sacrificing detection completeness.

### 2.5. Adaptive Control System

#### 2.5.1. Architecture

The control system is split between two devices. The high-level control logic runs on the inference and analysis node alongside the YOLO26 pipeline and produces a command at a sampling cadence of $T_{s}=0.5$ s. The low-level PWM signal generation runs on an **Arduino microcontroller** which receives the command from the host via USB serial and updates the PWM output to the mill actuators. This separation isolates the deterministic low-level signal generation from the variable scheduling latency of the host operating system.

#### 2.5.2. Quality Metric

The instantaneous broken-rice percentage B(k) defined in Section 2.3 is the primary controlled variable. To attenuate frame-level stochastic noise, a 10-sample moving-average filter is applied:

(2) $$\bar{B}\left(k\right)=\frac{1}{N}\sum_{i=0}^{N-1}B\left(k-i\right),\;N=10.$$

The window corresponds to 5 s of conveyor throughput and was selected to balance noise rejection against control bandwidth.

#### 2.5.3. Control Law

A discrete-time proportional (P) feedback law is implemented:

(3) $$u\left(k\right)=u_{0}+K_{p}\left[\bar{B}\left(k\right)-B_{sp}\right],$$

where $u_{0}$ is the nominal duty cycle, $K_{p}$ is the proportional gain, and $B_{sp}=8\%$ is the broken-rice setpoint, chosen to coincide with the upper bound of the Grade A commercial specification used in major rice-trading markets. The output is saturated to the admissible PWM range (Section 2.5.4).

Justification for P (rather than PI or PID).

A proportional-only law was adopted for the first-generation prototype for three reasons: (i) the controlled variable $\bar{B}\left(k\right)$ is a fraction, not a regulated absolute quantity, so steady-state offset is tolerated by the operational acceptance criterion; (ii) the integral term risks windup under the narrow PWM saturation band described below; and (iii) the dominant process disturbances are oscillatory (grain-density fluctuations in the feed) rather than constant, reducing the marginal benefit of integral action. The gain $K_{p}$ was selected empirically through bench-top testing of stable closed-loop tracking across the operating range; the empirical plant gain identified in Section 3.5 now provides the quantitative basis for the formal PI/PID/MPC controller synthesis identified as priority future work (Section 5.2).

#### 2.5.4. Actuator Mapping and PWM Range

The PWM duty cycle is saturated to the band $\left[u_{\min},u_{\max}\right]=\left[1.5\%,4.0\%\right]$. This narrow range reflects two physical constraints: the lower bound is the minimum duty cycle below which the mill motor stalls under load, and the upper bound is the maximum duty cycle above which sustained mechanical loading exceeds the manufacturer-rated continuous-operation envelope of the vibrators. Within this band, the actuators modulate both the milling speed (via the motor) and the grain-flow vibration intensity (via the vibrators).

#### 2.5.5. Open-Loop Benchmarking Protocol

To isolate the contribution of the adaptive controller from the underlying milling and detection performance, a matched open-loop benchmark was conducted in which the PWM command was held at a fixed nominal value with no feedback from the vision pipeline. All other system parameters, grain population, feed rate, illumination, camera configuration, and the YOLO26 model, were held identical to those of the closed-loop experiment. The two configurations therefore differ in exactly one variable: the activation state of the closed-loop controller.

#### 2.5.6. Classifier Validation on Manual Annotations

To quantify the error contribution of the geometric-threshold post-process classifier independently of the closed-loop control task, a ground-truth corpus of 1635 kernels was constructed by manually annotating 17 images from the same experimental session with polygon segmentation. For each polygon, the length along the major axis, $\ell_{\mathrm{gt}}$, was computed by principal component analysis of the polygon vertices, giving the length of the oriented bounding rectangle in pixels. The paper’s Good/Broken definition (Section 2.1 was applied via a physical calibration factor of $k_{px}=0.1228$ mm/px, derived from the median whole-kernel length in the pipeline operating data (57 px) and a typical *Oryza sativa* long-grain reference length of 7 mm, giving a 5.5 mm equivalent of $L_{\mathrm{gt}}=45$ px. A sensitivity check across $L_{\mathrm{gt}}\in \left[40,48\right]$ px confirmed that all reported metrics vary by less than 2 percentage points across this range.

Ground-truth labels were assigned according to

(4) $$c_{\mathrm{gt}}\left(k\right)=\left\{\begin{array}{cc} \mathrm{Good} & \mathrm{if}\;\ell_{\mathrm{gt}}\left(k\right)\geq L_{\mathrm{gt}},\\ \mathrm{Broken} & \mathrm{otherwise}, \end{array}\right.$$

consistent with the paper’s stated 5.5 mm length rule. The geometric-threshold post-process classifier, matching the paper’s stated aspect-ratio-and-contour-area formulation, was then applied to the same polygons using

(5) $$c_{\mathrm{pred}}\left(k\right)=\left\{\begin{array}{cc} \mathrm{Good} & \mathrm{if}\;r\left(k\right)>r_{th}\;\mathrm{and}\;A\left(k\right)\geq A_{th},\\ \mathrm{Broken} & \mathrm{otherwise}, \end{array}\right.$$

where r(k)=ℓ(k)/w(k) is the aspect ratio, A(k) is the polygon area, $r_{th}=2.0$, and $A_{th}=440$ px^2^. Both classifier thresholds were established from the natural distribution of the ground-truth polygon features, cross-checked against the operational values in the pipeline output. Agreement between $c_{\mathrm{gt}}$ and $c_{\mathrm{pred}}$ was reported as a kernel-level confusion matrix and, for the frame-level control signal, as a Bland–Altman analysis of the per-image broken-fraction agreement. This benchmark is not equivalent to gravimetric validation by sieving or weighing (a limitation acknowledged in Section 4.4) but is sufficient to bound the classifier’s contribution to the total measurement error.

## 3. Results

### 3.1. Detection Model Performance

**Figure 4:**
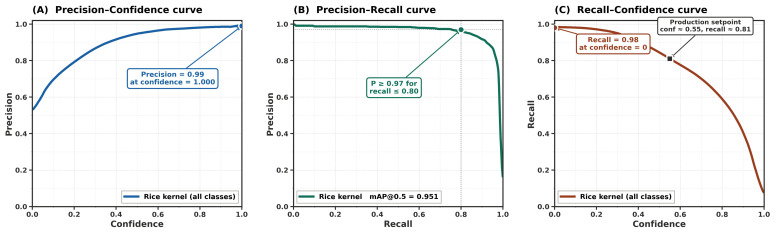
Performance evaluation of the YOLO26 model: (**A**) precision–confidence curve, (**B**) precision–recall curve, and (**C**) recall–confidence curve for rice kernel detection.

The fine-tuned YOLO26 detector achieved a mean Average Precision (mAP@0.5) of 0.951 on the held-out test set, with a peak precision of 0.99 and a peak recall of 0.98 (Figure 4). Precision exceeds 0.97 for recall up to approximately 0.80, and the steep precision decline at recall >0.90 reflects the most challenging partially occluded grains in the dense-feed condition. The F1 score peaks at 0.91 at a confidence threshold of 0.353, consistent with the operating threshold of 0.55 used in live operation.

**Figure 5:**
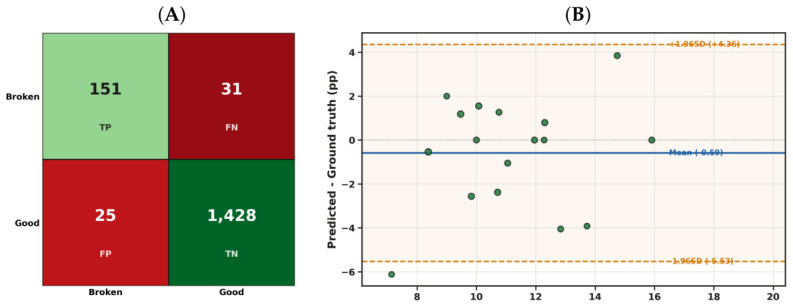
Classifier validation on the manually annotated ground-truth corpus. (**A**) Kernel-level confusion matrix, (**B**) Bland-Altman plot of the per-image broken-fraction agreement.

*Classifier validation:* On the 1635-kernel ground-truth corpus described in Section 2.5.6 and in Figure 5, the geometric-threshold post-process classifier achieved a kernel-level accuracy of 96.6%, with a broken-class precision of 85.8%, recall of 83.0%, and F1 score of 84.4%. Per-image broken-fraction estimates from the pipeline agreed with ground-truth per-image broken counts to within a mean absolute error of 1.84 pp, with a mean bias of −0.59 pp (slight under-report of broken fraction) and 95% Bland-Altman limits of agreement of [−5.53,+4.36] pp. This error bound is 8.1× smaller than the 14.85 pp closed-loop effect reported in Section 3.4.

### 3.2. Open-Loop (Baseline) Operation

**Figure 6:**
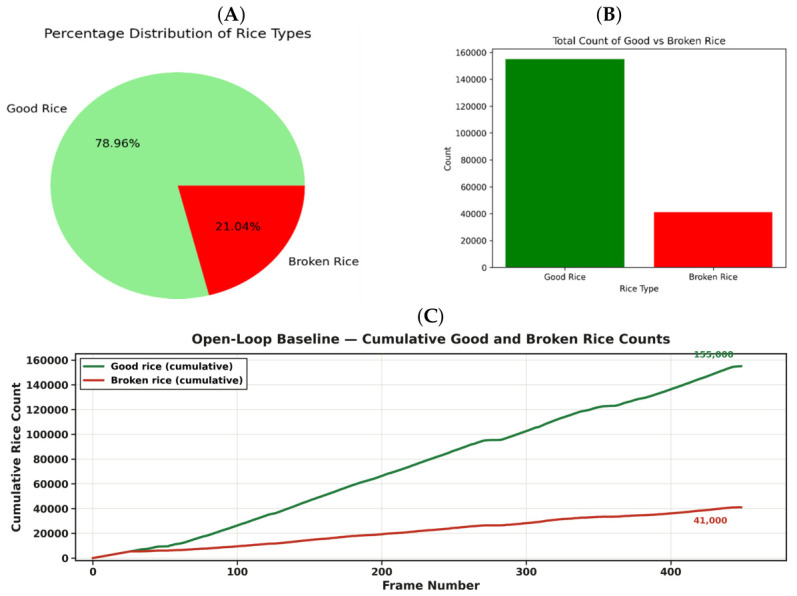
Open-loop (baseline) operation. (**A**) Class distribution: Good 78.96%, Broken 21.04%. (**B**) Total kernel counts, (**C**) cumulative Good (green) and Broken (red) counts vs. frame index, showing linear accumulation.

The open-loop benchmark session processed 196,000 kernels (155,000 Good, 41,000 Broken) over ≈440 consecutive frames. The overall Good/Total ratio was 78.96%, with a broken fraction of 21.04%. Cumulative kernel counts accumulated linearly with frame index, characteristic of constant-input operation with no internal correction for intermittent quality excursions (Figure 6).

### 3.3. Closed-Loop Operation

**Figure 7:**
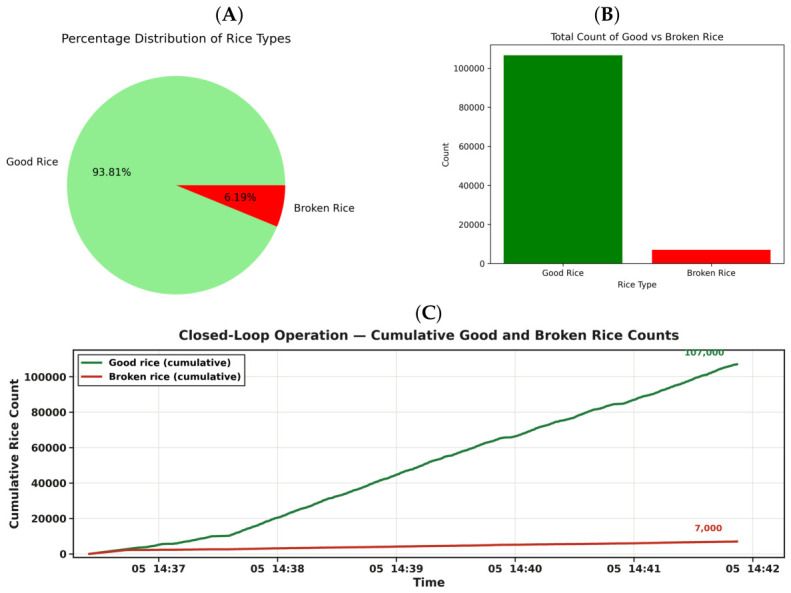
Closed-loop operation. (**A**) Class distribution: Good 93.81%, Broken 6.19%. (**B**) Total kernel counts. (**C**) Cumulative counts.

Following activation of the controller, a matched session processed 114,000 kernels (107,000 Good, 7000 Broken) over 465 frames at an average throughput of approximately 350 good grains per second. The Good/Total ratio rose to 93.81% with a broken fraction of 6.19% (Figure 7A–C).

**Figure 8:**
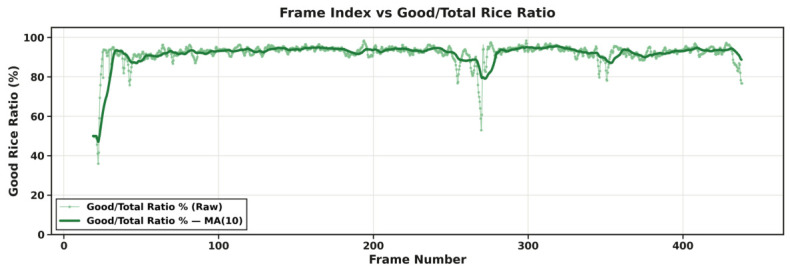
Per-frame Good/Total ratio (%); raw values shown lightly, 10-frame moving average shown bold.

The per-frame quality trace (Figure 8) shows a brief startup transient (frames 0–25) followed by sustained operation above 88% Good/Total, with two transient quality excursions near frames 250–265 and 360–380, from which the controller recovered within approximately 15 frames.

### 3.4. Comparative Analysis: Open-Loop vs. Closed-Loop

The matched comparison is summarised in Table 1. Closed-loop operation increased the Good/Total ratio by 14.85 percentage points and reduced the broken fraction by 70.58% relative to baseline. A two-proportion *z*-test confirms the difference at all conventional significance thresholds: z=112.8, p<0.001, with 95% confidence interval for the absolute reduction of 14.62–15.08 pp. Additional concordance tests (chi-square, Cohen’s *h*, sample-size adequacy analysis) yield consistent conclusions and are reported in Appendix A to avoid distracting from the principal effect.

### 3.5. Dynamic Behaviour of the Closed-Loop Control Signal

**Figure 9:**
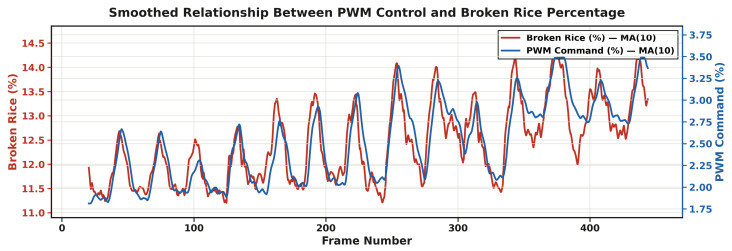
Dynamic correlation between PWM command (blue, right axis) and broken-rice percentage (red, left axis).

Figure 9 shows the time-aligned evolution of the smoothed PWM command and broken-rice percentage. The PWM oscillates within the band 1.9–3.7% across more than ten complete cycles within the 465-frame window, with the broken fraction ranging from 11.0% to 14.8% and showing near-in-phase co-movement with PWM at the cycle level.

Plant gain identification.

The empirical plant gain was estimated by linear regression of the cycle-peak broken-rice values against the corresponding cycle-peak PWM values across all identified PWM-breakage cycles (n=11 matched peak pairs), yielding:

(6) $$K^{*}=\frac{\Delta B}{\Delta u}\;\approx \;1.0–1.5\%\mathrm{broken}\mathrm{per}1\%\mathrm{PWM},$$

with a strong linear fit across the operating band. The narrow range reflects mild nonlinearity at the actuator extremes; within the operating band, $K^{*}$ is approximately constant. This identified gain is the principal empirical contribution of this work for control-systems design, since it specifies the open-loop plant transfer characteristic that any future PI/PID/MPC synthesis must compensate for.

## 4. Discussion

### 4.1. Detection Performance in Context

The achieved mAP@0.5 of 0.951 is broadly comparable with the best previously reported single-class rice-kernel detection performance, which lies in the range 0.92–0.96 for recent CNN- and transformer-based approaches [4,7,17]. The contribution of the present work is therefore not detection accuracy per se but the demonstration that detection of this quality is sufficient to drive a stable closed-loop control regime. The 0.99 peak precision is particularly relevant for control applications because it bounds the worst-case false-positive contamination of the control signal $\bar{B}\left(k\right)$, which would otherwise propagate as actuator chatter.

### 4.2. Quantified Impact of Closed-Loop Integration

The 14.85 pp absolute reduction in broken-rice fraction (70.58% relative reduction) is substantially larger than typical single-intervention gains reported in the rice-milling and broader food-processing literature, where 2–5 pp improvements are common for individual process upgrades [2,13,18]. The magnitude of the effect, combined with its statistical robustness and the matched experimental design, supports the interpretation that the observed change is causally attributable to the closed-loop controller rather than to confounding variation.

The mechanism is mechanistically interpretable. Under fixed-PWM operation, the mill applies a constant mechanical-loading regime that on average exceeds what is required for the prevailing grain density. Stochastic increases in grain density, arising from normal hopper-feed variation, are then translated directly into increased collision frequency and elevated breakage. Under closed-loop operation, the controller observes the consequent rise in $\bar{B}\left(k\right)$ and reduces PWM to lower mechanical loading precisely when required, operating closer to the throughput quality Pareto frontier than any fixed operating point can achieve.

### 4.3. Plant Dynamics and Implications for Controller Synthesis

The identified plant gain $K^{*}\approx 1.0$–1.5% breakage per 1% PWM has two immediate engineering implications. First, the linearity of the relationship within the operating band confirms that traditional linear controller-design techniques (PI, PID, linearised MPC) are applicable; non-linear or gain-scheduled controllers are not required for the present operating range. Second, the magnitude of $K^{*}$ implies meaningful actuation authority: a 1% reduction in mean PWM should yield a 1.0–1.5% reduction in mean broken fraction, which would push the closed-loop steady state from 6.19% comfortably into Grade A territory (<5%) without further hardware modification.

### 4.4. Deployment Considerations and Limitations

The current system is a laboratory-scale demonstrator and several constraints must be acknowledged before any industrial deployment claim can be supported:

1. **Scale:** The R.I.B. is a single-lane inspection bed operating at ∼350 Good grains/s. Industrial mills operate at throughputs that are orders of magnitude higher; multi-lane vision and parallel actuation would be required.
2. **Single-cultivar validation:** All training, validation, and milling experiments were conducted on a single rice cultivar under fixed moisture content and standardised laboratory illumination. Performance under different cultivars, moisture levels, dust loadings, and lighting conditions has not been validated in this study, and the reported quantitative results should be interpreted as characterising the plant under the tested conditions rather than as directly transferable to industrial deployment.
3. **No industrial trials:** The system has not been deployed in a working mill, and the closed-loop benefit reported here may attenuate under the broader disturbance spectrum of an industrial environment (load variation, dust, and vibration of upstream equipment).
4. **Operational limitations:** The current P-only control law has no formal stability proof or quantitative performance metrics (rise time, overshoot, settling time). These are reported as priority future work below.
5. **No gravimetric validation:** Quality estimation in this study is derived from vision-based detection with post-processing; comparison against gold-standard gravimetric sieving or weighing methods was not performed. While classifier-level validation against manual kernel labels (Section 2.5.6) bounds the vision-pipeline error, formal instrument-comparison studies against physical reference methods remain necessary before any industrial certification claim.

A pragmatic roadmap to industrial deployment would proceed in three stages: (a) extended laboratory validation across rice varieties and moisture conditions; (b) a pilot-scale trial on a single production line in partnership with a commercial miller, with formal controller redesign (PID or linearised MPC using the identified $K^{*}$) and full controller performance characterisation; and (c) multi-line industrial rollout with edge inference on Jetson-class hardware.

### 4.5. Latency Budget of the Cloud-Mediated Architecture

**Figure 10:**
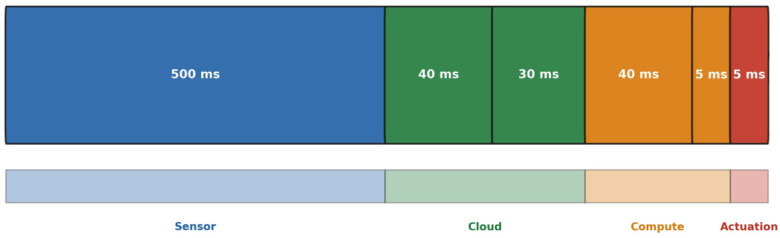
Component-wise latency budget of the closed-loop round-trip.

The cloud-mediated architecture introduces variable network latency that must be small compared with the closed-loop sampling period $T_{s}=0.5$ s for the P controller to remain effective. Per-stage latency was not formally instrumented in the present study; Figure 10’s component-wise engineering estimate is nonetheless useful for interpretation. Frame capture at 2 fps introduces a nominal 500 ms between successive frames. Upload of a single JPEG compressed frame (approximately 500 kB) over a stable ethernet connection typically completes in tens of milliseconds. YOLO26 inference on CPU is reported by [15] to complete in the tens of milliseconds for the nano variant. Command transmission to the microcontroller over USB serial and PWM updates are essentially instantaneous relative to these scales. The observation that the closed-loop system sustains stable tracking across more than ten complete PWM-breakage cycles (Figure 9) is direct experimental evidence that the total round-trip latency falls within the acceptable range for the plant’s dynamics under the tested conditions. Formal per-stage latency characterisation and industrial jitter analysis are identified as priority future work (Section 5.2).

## 5. Conclusions and Future Work

### 5.1. Conclusions

This study has demonstrated, on a laboratory-scale prototype and against a matched open-loop baseline, that a vision-guided proportional feedback loop can produce a substantial reduction in rice-milling breakage at constant throughput under the specific tested conditions. The 14.85 pp absolute reduction in broken-rice fraction observed under closed-loop operation moves the system from non-compliant to within the upper Grade A specification under those conditions, with an improvement of 70.58% relative to baseline that is statistically robust at p<0.001 and supported by an effect size of h≈0.45. Beyond demonstrating that such an architecture can be built, the work makes two specific scientific contributions. First, it establishes that detection of the quality achieved by YOLO26 (mAP@0.5 = 0.951) is operationally sufficient to support stable closed-loop control and a non-trivial result, since control-loop stability requires not just average accuracy but bounded false-positive contamination of the feedback signal. Second, by identifying the plant gain $K^{*}\approx 1.0$–1.5% breakage per 1% PWM, it provides the empirical transfer characteristic required for principled future controller design.

### 5.2. Future Work

Four priority directions follow from the present work:

1. **Formal controller synthesis:** Using the identified $K^{*}$, design and tune PI, PID, and linearised MPC controllers with explicit stability analysis (gain and phase margin, closed-loop pole placement) and quantitative performance metrics (rise time, settling time, overshoot, steady-state error), as recently reported for agricultural and motor-control applications [19,20].
2. **Multi-varietal generalisation:** Extend the training dataset to cover multiple rice varieties, moisture contents, and lighting environments, and quantify domain-adaptation requirements.
3. **Edge deployment:** Port the YOLO26 model to embedded edge-inference platforms (Jetson Orin Nano, Xavier NX) to remove the cloud dependency for sub-second latency and lower deployment cost.
4. **Pilot-scale industrial trial:** Deploy the architecture on a working industrial milling line, with industry-partner co-design of multi-lane sensing and parallel actuation, and characterise the Grade-A premium economic return under realistic disturbance conditions.
5. **Comparative controller evaluation:** Conduct a systematic quantitative comparison of P, PI, and PID controllers using the identified plant gain $K^{*}$, under matched disturbance conditions, with explicit reporting of rise time, settling time, overshoot, steady-state error, and disturbance rejection metrics, together with formal stability analysis (gain and phase margin, closed-loop pole placement).
6. **Comparative classifier evaluation:** Benchmark the geometric-threshold post-process classifier against lightweight CNN and segmentation-based classifiers to quantify the trade-off between auditability and detection performance.
7. **Gravimetric validation:** Conduct an instrument-comparison study against gold-standard gravimetric sieving and weighing methods to formally certify the vision pipeline for industrial quality assurance.
8. **End-to-end latency characterisation:** Provide formal per-stage latency instrumentation of the cloud-mediated pipeline, together with a comparative evaluation against edge-only deployment, to quantify the architectural trade-off.
9. **Multi-class quality control:** Extend the closed-loop objective from a two-class (Good/Broken) formulation to a multi-class quality space including pigmentation-based categories (e.g., brown rice, chalky rice), with a corresponding multi-objective or weighted control law.

## Figures and Tables

**Table 1:** Comparative performance under open-loop and closed-loop operating regimes.

Metric	Open-Loop	Closed-Loop	Change
Total kernels processed	196,000	114,000	—
Good kernels	155,000	107,000	—
Broken kernels	41,000	7000	—
Good fraction (%)	78.96	93.81	+14.85 pp
Broken fraction (%)	21.04	6.19	−14.85 pp
Relative breakage reduction (%)	—	—	−70.58
Grade A compliance (<5–8% broken)	Not met	Met	—

## Data Availability

The YOLO26 detection output dataset, classification and analysis results generated in this study are available from the corresponding author upon reasonable request at h.zhang@shu.ac.uk.

## Abbreviations

The following abbreviations are used in this manuscript:

COCO	Common Objects in Context (benchmark dataset)
DFL	Distribution Focal Loss
F1	Harmonic mean of precision and recall
fps	Frames per second
IoU	Intersection over Union
mAP	Mean Average Precision
MPC	Model Predictive Control
NMS	Non-Maximum Suppression
P	Proportional (controller)
PI	Proportional-Integral (controller)
PID	Proportional-Integral-Derivative (controller)
pp	Percentage points
PWM	Pulse-Width Modulation
R.I.B.	Rice Inspection Bed
YOLO	You Only Look Once

## Appendix A. Supplementary Statistical Analyses

Chi-square test:

A chi-square test of independence on the 2×2 contingency table of outcomes (Good/Broken) across regimes yielded $\chi^{2}=12,714$ (df=1, $p<10^{-15}$), confirming that the regime effect is not attributable to sampling variation.

Effect size:

Cohen’s *h* for the two proportions:

$$h=2\;|\arcsin\sqrt{0.9381}-\arcsin\sqrt{0.7896}|\approx 0.455,$$

corresponding to a medium effect by conventional Cohen criteria.

Statistical power:

At α=0.05, power 1−β=0.80, and the realised sample sizes ($n_{1}=196,000$, $n_{2}=114,000$), the minimum detectable difference in proportions is approximately 0.3 percentage points, two orders of magnitude smaller than the observed difference. The test is therefore very substantially overpowered.

## References

[1] Khush G.S. (2005). What it will take to feed 5.0 billion rice consumers in 2030. Plant Mol. Biol..

[2] Ilo B., Badjona A., Singh Y., Shenfield A., Zhang H. (2025). Artificial intelligence in rice quality and milling: Technologies, applications, and future prospects. Processes.

[3] Sultana S., Faruque M., Islam M.R. (2022). Rice grain quality parameters and determination tools: A review. Int. J. Food Prop..

[4] Suma D., Narendra V.G., Holla M.D., Holla M.R. (2025). Intelligent rice quality assessment using hybrid CNN-clustering approach. Discov. Appl. Sci..

[5] Siddique A., Gupta A., Sawyer J.T., Huang T.S., Morey A. (2025). Big data analytics in food industry: A state-of-the-art literature review. npj Sci. Food.

[6] Ilo B., Rippon D., Singh Y., Shenfield A., Zhang H. (2025). Real-Time Rice Milling Morphology Detection Using Hybrid Framework of YOLOv8 Instance Segmentation and Oriented Bounding Boxes. Electronics.

[7] Kurniawan H., Arief M.A.A., Manggala B., Kim H., Lee S., Kim M.S., Baek I., Cho B.K. (2025). Development of an intelligent inspection system based on YOLOv7 for real-time detection of foreign materials in fresh-cut vegetables. Agriculture.

[8] Qiu Z., Wang F., Wang W., Li T., Jin X., Qing S., Shi Y. (2024). YOLO-SDL: A Lightweight Wheat Grain Detection Technology Based on an Improved YOLOv8n Model. Front. Plant Sci..

[9] Ilo B., Lwele E., Singh Y., Zhang H. (2025). Classification and Morphology Detection of Rice Using Machine Vision and Deep Learning. International Symposium on Measurements and Control in Robotics.

[10] Zia H., Fatima H.S., Khurram M., Hassan I.U., Ghazal M. (2022). Rapid Testing System for Rice Quality Control through Comprehensive Feature and Kernel-Type Detection. Foods.

[11] Fan F., Chen H., Gao Y., Mou T. (2024). Quantitative Detection and Sorting of Broken Kernels and Chalky Grains in Milled Rice Using Computer Vision Algorithms. J. Food Eng..

[12] Kroičs K., Būmanis A. (2024). BLDC Motor Speed Control with Digital Adaptive PID-Fuzzy Controller and Reduced Harmonic Content. Energies.

[13] Maślanka M., Jancarczyk D., Rysinski J. (2025). Integration of machine vision and PLC-based control for scalable quality inspection in Industry 4.0. Sensors.

[14] Ultralytics YOLO26—Ultralytics YOLO Docs. https://docs.ultralytics.com/models/yolo26/.

[15] Sapkota R., Cheppally R.H., Sharda A., Karkee M. (2025). YOLO26: Key architectural enhancements and performance benchmarking for real-time object detection. arXiv.

[16] Hua Z., Aranganadin K., Yeh C.-C., Hai X., Huang C.Y., Leung T.C., Hsu H.Y., Lan Y.C., Lin M.C. (2025). A benchmark review of YOLO algorithm developments for object detection. IEEE Access.

[17] Li Z., Wu W., Wei B., Li H., Zhan J., Deng S., Wang J. (2025). Rice Disease Detection: TLI-YOLO Innovative Approach for Enhanced Detection and Mobile Compatibility. Sensors.

[18] Alejandro-Sanjines U., Maisincho-Jivaja A., Asanza V., Lorente-Leyva L.L., Peluffo-Ordóñez D.H. (2023). Adaptive PI Controller Based on a Reinforcement Learning Algorithm for Speed Control of a DC Motor. Biomimetics.

[19] Huang M., Tian M., Liu Y., Zhang Y., Zhou J. (2022). Parameter Optimization of PID Controller for Water and Fertilizer Control System Based on Partial Attraction Adaptive Firefly Algorithm. Sci. Rep..

[20] Jha K., Doshi A., Patel P., Shah M. (2019). A Comprehensive Review on Automation in Agriculture Using Artificial Intelligence. Artif. Intell. Agric..

